# Repurposing of Omarigliptin as a Neuroprotective Agent Based on Docking with A_2A_ Adenosine and AChE Receptors, Brain GLP-1 Response and Its Brain/Plasma Concentration Ratio after 28 Days Multiple Doses in Rats Using LC-MS/MS

**DOI:** 10.3390/molecules26040889

**Published:** 2021-02-08

**Authors:** Bassam M. Ayoub, Haidy E. Michel, Shereen Mowaka, Moataz S. Hendy, Mariam M. Tadros

**Affiliations:** 1 Pharmaceutical Chemistry Department, Faculty of Pharmacy, The British University in Egypt, El-Sherouk City, Cairo 11837, Egypt; shereen.hassib@bue.edu.eg (S.M.); moataz.sobhy@bue.edu.eg (M.S.H.); 2 The Center for Drug Research and Development (CDRD), Faculty of Pharmacy, The British University in Egypt, El-Sherouk City, Cairo 11837, Egypt; 3 Pharmacology and Toxicology Department, Faculty of Pharmacy, Ain Shams University, Organization of African Unity Street, Abassia, Cairo 11566, Egypt; heidieffat@pharma.asu.edu.eg; 4 Analytical Chemistry Department, Faculty of Pharmacy, Helwan University, Ain Helwan, Cairo 11795, Egypt; 5 Pharmaceutical Analytical Chemistry Department, Faculty of Pharmacy, Ain Shams University, Organization of African Unity Street, Abassia, Cairo 11566, Egypt; mariam.tadros@pharma.asu.edu.eg

**Keywords:** repurposing, omarigliptin, docking, A2A adenosine receptor, acetylcholine esterase receptor, GLP-1, rats’ plasma, rats’ brain tissue, 28 days multiple doses, LC-MS/MS

## Abstract

The authors in the current work suggested the potential repurposing of omarigliptin (OMR) for neurodegenerative diseases based on three new findings that support the preliminary finding of crossing BBB after a single dose study in the literature. The first finding is the positive results of the docking study with the crystal structures of A_2A_ adenosine (A2AAR) and acetylcholine esterase (AChE) receptors. A2AAR is a member of non-dopaminergic GPCR superfamily receptor proteins and has essential role in regulation of glutamate and dopamine release in Parkinson’s disease while AChE plays a major role in Alzheimer’s disease as the primary enzyme responsible for the hydrolytic metabolism of the neurotransmitter acetylcholine into choline and acetate. Docking showed that OMR perfectly fits into A2AAR binding pocket forming a distinctive hydrogen bond with Threonine 256. Besides other non-polar interactions inside the pocket suggesting the future of the marketed anti-diabetic drug (that cross BBB) as a potential antiparkinsonian agent while OMR showed perfect fit inside AChE receptor binding site smoothly because of its optimum length and the two fluorine atoms that enables quite lean fitting. Moreover, a computational comparative study of OMR docking, other 12 DPP-4 inhibitors and 11 SGLT-2 inhibitors was carried out. Secondly, glucagon-like peptide-1 (GLP-1) concentration in rats’ brain tissue was determined by the authors using sandwich GLP-1 ELISA kit bio-analysis to ensure the effect of OMR after the multiple doses’ study. Brain GLP-1 concentration was elevated by 1.9-fold following oral multiple doses of OMR (5 mg/kg/day, p.o. for 28 days) as compared to the control group. The third finding is the enhanced BBB crossing of OMR after 28 days of multiple doses that had been studied using *LC*-MS/MS method with enhanced liquid–liquid extraction. A modified LC-MS/MS method was established for bioassay of OMR in rats’ plasma (10–3100 ng/mL) and rats’ brain tissue (15–2900 ng/mL) using liquid–liquid extraction. Alogliptin (ALP) was chosen as an internal standard (IS) due to its LogP value of 1.1, which is very close to the LogP of OMR. Extraction of OMR from samples of both rats’ plasma and rats’ brain tissue was effectively achieved with ethyl acetate as the extracting solvent after adding 1N sodium carbonate to enhance the drug migration, while choosing acetonitrile to be the diluent solvent for the IS to effectively decrease any emulsion between the layers in the stated method of extraction. Validation results were all pleasing including good stability studies with bias of value below 20%. Concentration of OMR in rats’ plasma were determined after 2 h of the latest dose from 28 days multiple doses, p.o, 5 mg/kg/day. It was found to be 1295.66 ± 684.63 ng/mL estimated from the bio-analysis regression equation. OMR passed through the BBB following oral administration and exhibited concentration of 543.56 ± 344.15 ng/g in brain tissue, taking in consideration the dilution factor of 10. The brain/plasma concentration ratio of 0.42 (543.56/1295.66) was used to illustrate the penetration power through the BBB after the multiple doses for 28 days. Results showed that OMR passed through the BBB more effectively in the multiple dose study as compared to the previously published single dose study by the authors. Thus, the present study suggests potential repositioning of OMR as antiparkinsonian agent that will be of interest for researchers interested in neurodegenerative diseases.

## 1. Introduction

**Figure 1:**
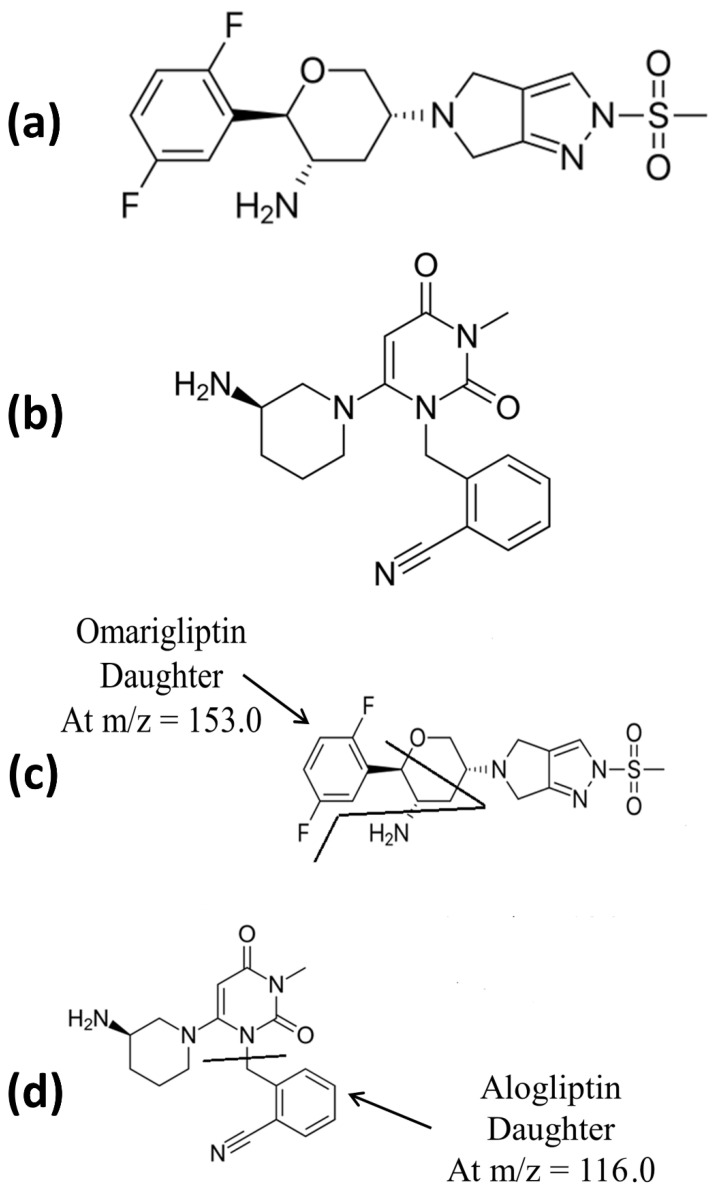
(**a**) Chemical structure of OMR, (**b**) chemical structure of IS, (**c**) daughter of OMR, and (**d**) daughter of IS.

Drug repurposing is a novel research topic as a substitute to underperforming hypothesis-driven molecular target-based drug discovery efforts [1]. De novo drug discovery is an outdated perspective that is pricey process. Thus, drug repurposing was a substitute strategy as therapeutic conversion of a drug that is already marketed is less time consuming [2]. It has proven to be a preferred approach for enhanced drug discovery that carries less risk due to the presence of previous pharmacological, safety, and toxicology data [3] with many positive studies in the literature [4,5]. Omarigliptin (OMR) is a novel once weekly anti-diabetic drug [6]. Dipeptidyl peptidase-4 (DPP-4) inhibitors are well-known effective potential agents against type 2 diabetes mellitus (DM). OMR is a long-acting once weekly administered DPP-4 inhibitor (Figure 1a), that prolongs the half-life of glucagon-like peptide-1 (GLP-1) and increases the insulin production [7,8,9,10,11,12,13,14,15,16,17] as a treatment against type 2 DM. The once-weekly OMR has the advantage of more patient compliance than the other daily-administered DPP-4 inhibitors. Unlike the classical once-daily DPP-4 inhibitors, once-weekly administration could boost the patients’ compliance [18]. Interestingly, this will depict a major outcome if the drug had been repositioned successfully for Parkinson’s disease (PD) or at least as a neuroprotective agent against other neurodegenerative diseases. PD is the most prevalent movement neuro-degenerative disease [19] and this study aims to develop new curative agents for PD achieving wide safety margin implementing repositioning. Repositioning of a once-weekly anti-diabetic drug for the management of PD will promote the patient’s adherence with potential financial privilege due to once-weekly dosing regimen. Indeed, the potential neuroprotective effect of GLP-1 had been previously reported owing to the promising antiparkinsonian effect of DPP-4 inhibitors (Gliptins, anti-diabetics) [20,21]. Many gliptins exhibited neuro-restorative effects in previous PD studies related to DPP-4 inhibition as sitagliptin, saxagliptin, linagliptin, and vildagliptin [22,23,24,25,26,27,28]. Additionally, a new study recommended repositioning of teneligliptin to brain conditions [29]. Drug repositioning represents the most novel approach in drug development due to its efficiency and lower economic burden. Drug repurposing is an uprising revolution of drug discovery that presents major and prominent privilege with already approved safer agents by scanning the current candidates.

Although a literature review shows that OMR pharmacokinetics factors and/or bioanalysis were studied in human [7,8,9,10,11,12,13,14] and rats [15,16,17] successfully according to the common FDA bioanalytical guidelines [30]. All articles considered only the plasma parameters through bio-analysis either human plasma or rats’ plasma. While only one article that was published by (Ayoub et al.) considered the bioanalysis of OMR in rats’ brain tissue [17]. However, that method [17] used only direct precipitation as an extraction technique for OMR using acetonitrile. Extraction was enhanced after using ethyl acetate as the extracting solvent accompanied with adding 1N sodium carbonate to enhance the drug migration. In addition, extraction was enhanced after decreasing any emulsion between the layers in the liquid–liquid extraction effectively using acetonitrile as the diluent solvent for the IS [31]. It is worth mentioning that the described extraction method was a slightly modified method applicable on both rats’ brain tissue and rats’ plasma samples and it had been firstly described and used for human plasma samples by Addy and Tatosian et al. [10,11,12,13]. As per FDA guidelines, the bio-analytical method validation was established to include linearity, precision, selectivity, accuracy, carry over, extraction recovery, stability studies, and matrix factor [30]. Alogliptin (ALP) was used as an internal standard (IS) due to its LogP value of 1.1, which is very close to the LogP of OMR. Extraction of OMR from both rats’ plasma and rats’ brain tissue samples was successfully achieved.

In the previously published article by (Ayoub et al.), it showed the preliminary OMR successful BBB crossing [17], direct precipitation followed by LC-MS methods were performed for determination of OMR and trelagliptin in rats’ plasma and brain tissue (after single dose of 5 mg/kg of OMR and 20 mg/kg of trelagliptin) to show their interaction with BBB to check for the opportunity of their repositioning as neuroprotective agents. Trelagliptin showed negative results while OMR crossed BBB successfully after the single dose. The proposed advanced current repositioning study of OMR in the underlying article is based on the docking study, the enhanced GLP-1 concentration in brain after the multiple dose study and finally the brain/plasma concentration ratio after multiple doses (28 days). Pharmaceutical industry and researchers working in PD treatment will highly benefit from this study. As per FDA guidelines [30], a complete validation of the new LC-MS/MS methods (that based on liquid–liquid extraction and vacuum evaporation for rats’ plasma and brain tissue) was developed. The validated methods will be suitable to researchers and QC laboratories for further pharmacokinetic and clinical studies. The new methods can also be used for further future studies (CDRD-BUE) calculating AUC_brain_/AUC_plasma_ after single and/or multiple dose pharmacokinetics (I.V., oral, intranasal, nanoformula to increase BBB crossing, etc.) in case of positive anti-parkinsonian results with different dose/response studies. Liquid–liquid extraction was common in the literature for extraction of many drugs from both animal plasma and brain extract [32,33,34] with high sensitivity (to overcome the limitation of high LLOQ in the pilot study with direct precipitation [17]). Working on the concentration ratio (brain/plasma) after multiple dose administration to rats (28 days), will offer interesting data about the behavior of OMR crossing the BBB on the long-term treatment plan.

## 2. Methods

### 2.1. Docking Study of OMR, Other 12 DPP-4 Inhibitors and 11 SGLT-2 Inhibitors with A_2A_ Adenosine Receptor (A2AAR) and Acetylcholinesterase (AChE) Receptor

OMR was docked into the crystal structures of A2AAR and AChE receptors. Moreover, for a computational comparative study of OMR docking involving 12 DPP-4 inhibitors and 11 SGLT-2 inhibitors was carried out. The 3D receptors’ structures were downloaded from Protein Data Bank (Codes: 3PWH and 6F25, respectively), then their preparation was implemented using MakeReceptor app of OpenEye Scientific Software tools. Different gliptins (DPP-4 inhibitors) and gliflozins (SGLT-2 inhibitors) were studied separately, and their energy were minimized using Open Babel software applying MMFF94 molecular force field. The docking calculations were proceeding on the protein model by FRED docking app of OpenEye.

### 2.2. Chemicals and Reagents

Various batches of blank rats’ plasma and rats’ brain tissue homogenate (10%) were prepared directly before the experimental work in the Center for Drug Research and Development at the British University in Egypt (CDRD-BUE). All the plasma and brain tissue samples were withdrawn as fresh samples in the animal house unit (CDRD) after the approval of the ethical committee (BUE). OMR raw material (99.0%), the raw material of alogliptin, which was used as IS, was certified to contain (99.2%) ethyl acetate were a generous gift thankfully provided by (CDRD, BUE). Acetonitrile, water, and methanol of HPLC grade and formic acid were acquired from (Sigma, USA). Sandwich ELISA kit (CUSABIO, CSB-E08117r) was used for GLP-1 estimation in rats’ brain tissue samples. The surfactant was used as 2.5% (*w*/*v*) aqueous solutions: Tween-80 was purchased from (El-Nasr Pharmaceutical Chemicals Co., Cairo, Egypt) in addition to sodium carbonate.

### 2.3. Biological Samples after Ethical Approval, Determination of Brain GLP-1 Concentration, and Multiple Dose In Vivo BBB Crossing Test

All experimental protocols and underlying methods were approved by the ethical committee of faculty of Pharmacy The British University in Egypt. Twenty rats (250 g ± 35) were randomly assigned into two groups (*n*=10); the first group was administered OMR (5 mg/kg/day, p.o.) for 28 days while the second group served as control. Rats’ dose was calculated in accordance with the FDA guidelines for human-rodent dose conversions [35]. GLP-1 brain concentration was assessed in both experimental groups. Also, LC-MS/MS was used for quantitative determination of OMR in plasma and brain tissue after oral administration to rats (*n*=10). The design of the study is a multiple dose study for 28 days based on 5 mg/kg/day, p.o. It is worth noting that tween 80 surfactant (2.5%, *w*/*v*) was essential to solubilize the hydrophobic OMR in saline (p.o). After 2 h of the latest dose after 28 days of OMR drug administration, 0.8 mL blood samples were collected into heparinized tubes via rats’ tail vein (except the control group). The separated plasma (>300 µL) was pipetted to clean tubes and stored at −80 °C until analysis. All twenty rats were then sacrificed and the whole brain of each animal was separated, washed in saline, homogenized (10%, *w*/*v* in saline) using Ultra-Turrax^®^ homogenizer and kept frozen at −80°C until LC-MS/MS analysis and GLP-1 ELISA kit analysis. The brain homogenate was centrifuged at 3000 rpm for 3 min then the supernatant was used for determination of GLP-1 concentration using Sandwich ELISA kit (CUSABIO, CSB-E08117r) according to a reported method [36]. Statistical analysis was performed using GraphPad Prism (version 7, ISI^®^ software, USA). GLP-1 results were expressed as the mean ± SEM and analyzed using two-tailed Student’s *t*-test test. Probability values lower than 0.05 were considered statistically significant. Also, the current study aimed to investigate whether the crossing ability of OMR for the BBB will be enhanced after the multiple dose for 28 days and to assess if the developed LC-MS/MS method is valid for the bioassay of OMR in the actual biological samples (plasma and brain tissue). The dilution factor of 10 was taken into consideration for all brain tissue calculations (LC-MS/MS and ELISA). 

### 2.4. LC-MS/MS Conditions

The following conditions were implemented based on the previously published work by Ayoub et al. after minor modifications to increase the sensitivity (published preliminary repositioning study on OMR using direct precipitation as an extraction technique for rats’ plasma [17] by Ayoub et al.). LC-MS/MS was performed via Waters^®^ UPLC-TQ with ESI, Mass Lynx software (4.2 version) and ACQUITY UPLC^®^ BEH shield RP C_18_ column (1.7 µm, 150 × 2.1 mm), (WATERS, Ireland, serial 01853004518304). Mobile phase of acetonitrile/0.3 formic acid (90:10, *v*/*v*) in the isocratic mode, 10 µL as the injection volume, 0.3 mL/min as the flow rate and 1.5 min as the run time for OMR bio-assay, were adopted. The mass spectrometer parameters included the adjustment of cone voltage values to 40 V and 30 V and collision energy values to 50 eV and 55 eV for OMR and alogliptin (IS), respectively. MRM of *m*/*z* equals 399.2 to 153.0 for OMR and *m*/*z* equals 340.2 to 116.0 for alogliptin IS in the ESI positive mode was applied (Figure 1). MS tuning was implemented and the stated parameters were adopted: turbo ion spray at 400 °C, capillary temperature at 275 °C, sheath gas at 15 psi, auxiliary gas at 2 psi, ion spray voltage at 5500 V, capillary voltage at 4 kV, capillary offset at 35, desolvating line temperature at 400 °C, source temperature at 130 °C, and desolvation gas flow at 600 L/h.

### 2.5. LC-MS/MS Calibrators, QC Samples, and Sample Preparation

OMR standard stock solution in methanol was prepared as (1 mg/mL) then working solutions were prepared using methanol as a solvent with multiple concentrations (1, 3, 5, 25, 40, 85, 150, 200, and 310 µg/mL). Ten microliters of each one of the prepared working solutions was used to spike 990 μL blank plasma to prepare calibrators as 10 ng/mL (LLOQ), 50, 250, 850, 1500, and 3100 ng/mL. QC samples were selected to be 30, 400, 2000 ng/mL as LQC, MQC, and HQC, respectively. While for brain homogenate samples (10%); working solutions with different concentrations were prepared in methanol (1.5, 3, 6, 15, 40, 60, 130, 200, and 290 µg/mL). A 10-microliter measure of each one of the prepared working solutions was used to spike 990 μL brain homogenate sample 10% to prepare calibrators as 15 ng/mL (LLOQ), 60, 150, 600, 1300, and 2900 ng/mL. QC samples were selected to be 30, 400, 2000 ng/mL as LQC, MQC, and HQC, respectively. For samples preparation, an aliquot of 300 μL of each plasma sample (or brain homogenate sample 10%) was spiked with 100 μL of the IS in acetonitrile (300 ng/mL), then 100 μL of 1 N sodium carbonate was added, followed by liquid–liquid extraction by 1.5 mL of ethyl acetate for 15 min centrifugation at 15,000 rpm. 1.3 mL of the upper organic level was vacuum evaporated until dryness followed by reconstituted with 300 μL methanol and then injected into the auto-sampler. Peak area ratios to IS was used against concentrations to estimate the calibration curve and the regression equation. The final brain samples’ concentrations were calculated as ng/g brain tissue after considering the dilution factor of 10.

### 2.6. LC-MS/MS Bioanalytical Validation

According to FDA guidelines [30] six calibrators were performed to estimate the linearity while LLOQ, LQC, MQC, HQC levels (*n* = 5) were determined to predict accuracy and precision five times a day and on three consecutive days. The final brain samples’ concentrations were calculated as ng/g brain tissue after considering the dilution factor of 10. The bias value, standard deviation (S.D.) and % RSD were estimated. Selectivity was determined by checking interference in six different batches of blank rats’ plasma and rats’ brain tissue homogenate (10%) that had been prepared at (CDRD-BUE) as mentioned under Section 2.2. Injection of blank samples directly after the high values of concentration was performed to confirm the lack of a carryover of a notable value. The matrix effect was estimated by comparing the area ratios under the peak of the post extracted samples to the neat standards. While the extraction recovery was estimated by comparing the area ratios under the peak of the underlying pre-extracted samples (spiked before extraction) to the post-extracted samples (spiked after extraction). The dilution integrity experiment for the rats’ plasma method was carried out at 5 times and 10 times dilution of the high concentration 3500 ng/mL and their concentrations were calculated, while for the brain homogenate experiment, it was carried out at 5 times and 10 times dilution of the high concentration 3000 ng/mL. Both high concentrations for the two experiments were higher than the upper limit of quantification. The percentage change from the comparison sample should be within ±15%. Stability of LOQ and HQC was determined based on four different bioassays including, leaving the samples in the auto-sampler for 3 h, leaving them at room temperature for 3 h (bench top short-term stability), analyzing them after three complete cycles of freeze and thaw, and finally, freezing the samples at −80 °C for two weeks was performed to inspect the long-term stability.

## 3. Results and Discussion

### 3.1. Comparative Docking Study (OMR, DPP-4 Inhibitors, and SGLT-2 Inhibitors) with A2AAR and AChE Receptors

#### 3.1.1. Comparative Docking Study of DPP-4 Inhibitors with A2AAR that Support Repurposing of OMR for Parkinson’s’ Disease

**Figure 2:**
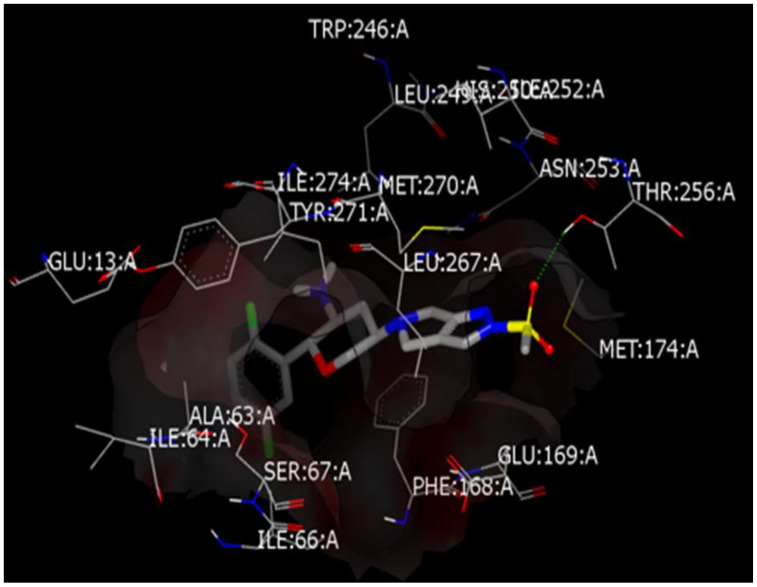
Predicted binding mode of OMR with A2AAR crystal structure.

Virtual simulation could be a guide in understanding activity or even anticipating certain pharmacological activity or analytical finding. Such a multifactorial disease like Parkinson’s, is affected by modulation of several proteins offering different treatment pathways and agents. It is considered that increasing the human body’s ability to control the blood sugar will result in better central and neurological functions. In addition, some recent DPP-4 inhibitors’ studies approved the achieving of central neuroprotective effects. In general, mediating certain activity via different mechanisms that has been coined poly-pharmacology would result in improved total efficacy. A docking simulation approach would enable identification of drugs into not kindred protein targets. Where is the study aiming to find out whether OMR after passing BBB would achieve central anti Parkinson activity or not, OMR was docked into the crystal structures of the A2A adenosine receptor (A2AAR) that is a member of non-dopaminergic GPCR superfamily receptor proteins and has essential role in regulation of glutamate and dopamine release. The crystal structure of the receptor that strongly enriched known ligands and has a comparably open binding site conformation was chosen (PDB code 3PWH). The co-crystallized ligand chemically named [4-{2-[(7-amino-2-furan-2-yl[1,2,4]triazolo[1,5-a][1,3,5]triazin-5 yl)amino]ethyl}phenol] was used to identify the active binding sites. Molecular docking calculations were achieved using OpenEye tools. At first, preparation of protein by MakeReceptor, energy minimization of OMR, generating all possible conformers and then docking via FRED. Docking shows that OMR is perfectly fit into binding pocket forming a distinctive hydrogen bond with Threonine 256, besides other non-polar interactions inside pocket, this shown in Figure 2 while Figure S1 in Supplementary Materials shows reliable score of the docking simulation with co-crystallized ligand. The difference in binding energy between the docked conformer of OMR and co-crystalized ligand in protein 3D structure was studied (as in Figure S1 in the Supplementary Materials) and the lower the energy of the conformer, the more predicted stability of the drug-receptor complex.

After the initial preliminary docking results of OMR, a computational comparison study of OMR docking and the other 12 DPP4 inhibitors was carried out. Utilizing the same non-dopaminergic member of the GPCR superfamily adenosine receptor A2A (A2AAR) was used which is considered as a pharmacologically alternative target for the antiparkinsonian active drugs [37,38]. The results were compatible with studies that utilized the same protein with the same Protein Data Bank code for testing new synthesized hits acting against Parkinson disease [39,40]. The 3D receptor structure was downloaded from Protein Data Bank (Code: 3PWH), then its preparation as implemented using MakeReceptor app of OpenEye Scientific Software tools [41,42,43]. Thirteen gliptins were studied separately, and energy minimized using Open Babel software applying MMFF94 molecular force field [44]. Subsequently, generation of all possible conformers of all the 13 drugs via OMEGA was possible [45]. The docking calculations were proceeding on the protein model by FRED docking app of OpenEye. All the 13 gliptins were fitted well in receptor pocket identified using the co-crystalized ligand. Two gliptins namely Alogliptin (Figure S2 in Supplementary Materials) and Linagliptin (Figure S3 in Supplementary Materials) scored a lower energy that suggest better affinity while fitting than the ligand after predicting the binding mode with 3PWH of each drug. Unfortunately, this better affinity does not ensure a better activity because of the lack of BBB crossing data about both alogliptin and linagliptin which is the main strength point for OMR. Furthermore, alogliptin made two hydrogen bonds with Asparagine 253 in binding site. In addition, teneligliptin, evogliptin, and anagliptin were found to hydrogen bond with asparagine 253, glutamic acid 169, and alanine 63, respectively. All of them showed perfect alignment inside the identified binding site. Figures S2 and S3 (in Supplementary Materials) confirmed the validation of docked structures inside the binding pocket achieving the anticipated effect. These figures do emphasize that if those gliptins crossed BBB, they do have the ability to fit in the desired receptor pocket achieving reasonable stable low energy complex. Docking scores are summarized in Table 1. Table 1 shows the docking energy result obtained from OpenEye. This score is of absolute value and it is directly related to the drug-receptor complex energy. The lower energy, the more stable the complex yet they all fit within the binding pocket. Other parameters may interfere like physical properties of each hit. In spite of OMR, not getting the highest score, to the latest of the authors knowledge, it is the only reported gliptin that crossed BBB—suggesting its higher exposure to the receptor in the brain.

#### 3.1.2. Off-Label Neuroprotective Effect of DPP-4 Inhibitors and SGLT-2 Inhibitors and How the Brain Is Insulin Dependent

A complicated chronic disease like type-2 diabetes mellitus (DM) is distinguished by its continuous need of monitoring to avoid harmful consequences. Main complications of DM includes dysfunction in ocular, nervous, cardiovascular, and renal systems. It results from insufficient insulin secretion or impaired tissue sensitivity to insulin or both [46]. Most recently, our research group studied two main classes of oral antidiabetic agents; namely, DPP-4 inhibitors which collectively known as gliptins and the other class are sodium glucose cotransporter-2 (SGLT2) inhibitors which also collectively known as gliflozins. Both are acting by indirect route affecting sugar level in blood. DPP-4 inhibitors lessen the breakdown of GLP-1, which is the major responsive glucose incretin hormone. The role of the unhydrolyzed GLP-1 intact hormones is to influence insulin secretion with the increase in blood glucose level. DPP-4 inhibitors are having lower potential to cause hypoglycemia as the resultant GLP-1 is secreted in case of high blood glucose level only [47,48]. SGLT2 inhibitors are located chiefly in the early proximal tubule in kidneys responsible for 80–90% of glucose reabsorption. By blocking their action most of reabsorbed glucose is then secreted in urine. This led to immediate lose in blood glucose without affecting any other signal mechanism [49].

The central effect of insulin has recently been proven in literature. This demonstrates why controlling blood glucose levels is in direct relation with better brain activities and cognitive functions [50]. In other words, the brain is an insulin responsive organ, studies include deletion of brain insulin receptors in mice results in hyperphagia, obesity, and dysfunction in insulin metabolic homeostasis [51]. Another recent study presented brain insulin resistance led to certain behavioral disorders and anxiety [52]. Generally, improper insulin signaling in the brain has a direct role in pathogenesis of insulin resistance occurs in DM, cognitive defects, and memory problems [53,54]. The case of impaired peripheral insulin production and sensitivity in DM not only results in complications and symptoms related to endocrine malfunctions, but also brain dysfunctions indicated by brain mitochondrial dysfunction, increased brain oxidative stress, impaired brain insulin receptor function, impaired synaptic plasticity, and overall cognitive decline [55,56,57].

This was validated by studies that shows direct or indirect changes in brain activity and functions when administering oral antidiabetics agents especially the most recently DDP-4 inhibitors. This class shows glycemia-independent advantageous outcomes in different studies adapting animal models of Parkinson’s disease and Alzheimer’s disease. However, there is no officially approved repositioned agent administered for a brain disorder. Yet ongoing and recent studies discuss the ability of other oral hypoglycemic agents for doing central therapeutic effects. Upon literature review of the recent antidiabetic agent that may play a double role in both DM and some cognitive disease, gliflozins appears to have a high potential [17,23,25,26].

SGLT2 receptors are also found in human brain doing the function of giving away glucose. A study of regional distribution of SGLT in rat brain, showed its presence in forebrain (in the caudate nucleus putamen and frontal cortex) and Midbrain (in the hippocampus, hypothalamus, parietal cortex, amygdala, and thalamus) [58]. Some studies of different animal models support this proposed role of SGLT2 inhibitors in elevating and promoting cognitive effects. A very recent model of Alzheimer’s disease on mixed murine rats, showed the impact of one of gliflozins; empagliflozin in decreasing vascular defect and cognitive deterioration. Long-term treatment with empagliflozin largely preserve memory and learning abilities of diabetic rats. Moreover, some cognitive improvement is resulted by a reduction of oxidative stress markers, better insulin signaling, and increased synaptic activity in the hippocampus [59]. Empagliflozin role seems to be related the attenuation of oxidative stress and reduction of cerebral superoxide and 8-OHdG. Attenuation of cerebral oxidative stress is mainly connected to a reduction in cerebral NADPH oxidase subunits, gp91phox and p67phox levels. Moreover, empagliflozin directly affect cerebral BDNF [60], a key protein promoting memory and survival of neurons, that is significantly decreased in DM patients and its decline is associated with cognitive decline. Interestingly, empagliflozin treatment significantly increased cerebral BDNF levels [61]. Other cognitive changes in diabetic mice after empagliflozin treatment, includes improvement in mice performance in object discrimination tests, along with increased neurogenesis in the dentate gyrus and synaptophysin in the striatum oriens [62]. Another study assessed the neuroprotective activity of the SGLT2 inhibitor dapagliflozin in combination with the GLP-1 agonist liraglutide in dietary-induced diabetic mice. In all treatment groups, recognition memory was significantly improved brain histology demonstrated increased staining (number of immature neurons) in dentate gyrus and synaptophysin (synaptic density) in stratum oriens and stratum pyramidale [62]. In which SGLT protein receptors are highly normally expressed [58].

The main problem with testing gliflozins and gliptins in brain is the insufficient data for their ability of passing blood brain barrier. There are assumptions of presence of these antidiabetic agents centrally in brain and other opinions suppose not and its SGLT2 inhibition modulates the autonomic nervous system in the kidneys. Signals from the kidney may be transmitted to the brain via the autonomic nervous system, thereby changing specific setpoints. This is an attempt to correlate molecular findings to put more light on the role of insulin as a whole-body affecting agent. Furthermore, this helps clinically in providing the optimum management of DM and its cognitive complications by providing a better understanding of oral hypoglycemic behavior and the role of insulin in brain functions. Therefore, the following computational study was carried out to calculate the binding energy between all gliflozins and gliptins with isolated crystal structure of some brain receptors of cognitive disorders like AChE enzyme receptor, for Alzheimer’s disease and A2AAR that plays a role in Parkinson’s disease. This might provide data for knowing the central mechanism of action of DPP-4 and SGLT2.

More than 30 years ago, GLP-1 was proposed to be a possible target to cure DM [63], as its infusion lessen total body need to insulin. It was already known that insulin response for oral sugar ingestion is greater than that is of intravenous infusion [64]. This was termed “the incretin effect” and attributed to stimulation of insulin secretion by incretins [65]. All DPP-4 inhibitors have the same anti-diabetic effects. The monotherapy results in smaller HbA1c reductions than metformin, but in sum, they are clinically equivalent to sulfonylureas and thiazolidinediones, when they are in combination with metformin [66]. Moreover, combination with metformin was related directly to lower gastric adverse effects [66,67]. Both metformin and DPP-4 inhibitors are the best initial combination in case of elevated HbA1c levels and could be also used in with lower values of HbA1c. This was introduced lately by the abbreviation VERIFY (vildagliptin efficacy in combination with metformin for early treatment of type 2 diabetes) study [68,69].

On the other hand, SGLT2 inhibitors was initially developed over the occurrence of phlorizin, which is a natural glycoside of plant origin. Phlorizin, the phytochemical obtained from apple tree bark was found to inhibit SGLT activity. After that when the mechanism of glucose transport was determined, optimum understanding of SGLTs and their main functional characteristics was gained [70,71,72]. Meanwhile SGLT2 inhibitors manifested clinical supremacy regarding hyperglycemia, visceral adiposity, and body weight. In addition, they ameliorate other metabolic problems come in line with high blood pressure, risky lipid profile, or uric acid level [73]. Cardiovascular outcome trials (CVOTs) of SGLT2 inhibitors showed better outcomes in diabetic or even nondiabetic patients [74,75]. SGLT transporters are one of SLC5 gene family [76]. Genes in this family include sugar-transporting proteins, anions (SLCSAS and SLC5A8), vitamins (SLC5A6), choline (SLC5A7), and one glucose sensor (SLC5A4) [77,78]. The main glucose transporters are SGLT1 and SGLT2. However, both do the same function, but they differ widely in properties [79,80]. Normally, blood glucose is accumulated to the side of glomerular membrane through tubular lumen. About 150 g of glucose is being filtered each day. However, per usual, these glucoses readily reabsorbed [81,82,83]. Glucose reabsorption is done over two steps, firstly at the basolateral membrane of proximal convoluted tubule. Where both SGLT1 and SGLT2 actively transport glucose in an ATPase facilitated diffusion into tubular epithelium cells. Second stage is mainly derived via glucose itself, that passively pass to plasma via Glucose transporter 2 [GLUT2] [84]. It is worth mentioning that, generally, glucose reabsorption in DMT2 patients is higher than normal and that is because of SGLT1 and GLUT2 overexpression [85,86].

As a new aminopeptidase with novel unique characteristics, DPP-4 was discovered in 1966 [87]. In 1992, its gene was identified on the long arm of (2q24.3 chromosome 2) encoding protein of 766-amino acids [88,89,90]. It is a member of gene family knowns as serine peptidase/prolyl oligopeptidase, which is subclassified by function or structure [91]. This DPP-4 protein is vastly expressed in several body tissues from endothelial cells in many vascular beds [92], rendering its circulatory substrates found within kidney, liver, lung, and gut [93]. DPP-4 act by transmitting signals across cell membrane plus interconnecting with other proteins in membrane. Curiously, the largest part of protein located extracellular and just six amino acids are merged into cell cytoplasm [37,94]. Meanwhile, a soluble form of DPP-4 is produced and liberated into plasma leading to its activity in human serum [95,96]. This active soluble form was found initially in saliva and serum then it had been detected in cerebrospinal fluid and bile [97]. More research is needed for understanding the mechanism by which DPP-4 starts a signal transduction cascade. It may occur through reaction with mannose 6-phosphate/IGF-2 receptor [98] or through other molecular interactions [99].

It is becoming obvious: brain is an insulin sensitive organ that controls many metabolic signal cascades [100]. Constant ATP supply in brain needs constant glucose uptake, which it turns controls general brain energetics. That is why the need for insulin emerges as a preventing factor for neurodegenerative disorders [101,102,103,104,105]. Insulin can enter the brain directly via blood–brain barrier (BBB) or indirectly via cerebrospinal fluid as a surrogate, in which insulin low concentrations transported through the endothelium locally in a region namely Virchow–Robin space [106]. Insufficient insulin led to impaired glucose uptake. Overtime, that would ruin ion transportation and recycling within vesicles heading to improper synaptic signaling within brain [107,108,109]. Inefficient perpetuation of ion gradients disrupts release of brain transmitters and a consecutive of adverse events would occurs including hyperexcitability, excitatory–inhibitory imbalance. These would all diminish the brain energy functions. These mentioned alterations result in worsening glutamatergic transmission and oligodendrocyte function and absolutely lead to total nutrient recycling reduction [110,111,112]. In fact, hypo-metabolism of glucose in brain has no obvious single cause. It may be out of reduction glucose uptake, a problem in aerobic glycolysis and in tricarboxylic acid cycle. Other factors may contribute, like inefficient axonal transport or decrease in glial energy support of the neurons [113,114].

#### 3.1.3. Docking Study of OMR (and 12 Other DPP-4 Inhibitors) with AChE Receptor

A computational comparison study of OMR and the other DPP-4 inhibitors (Table 1) was carried out after docking with AChE, which plays the main role in Alzheimer’s disease. This is come in line with studies utilized the same protein with the same Protein Data Bank code for testing new synthesized hits acting against Alzheimer’s diseases [49]. The 3D receptor structure was downloaded from Protein Data Bank (Code 6F25). Protein was initially prepared, using MakeReceptor app of OpenEye Scientific Software tools [50,51,52]. Thirteen DPP-4 inhibitors were studied and energy minimized using Open Babel software applying MMFF94 molecular force field [53]. Subsequently generation of all possible conformers of all via OMEGA [54]. The docking calculations were proceeding on the protein model by FRED docking app of OpenEye. DPP-4 inhibitors with AChE receptor adapt different binding behaviors due to lack of structure similarity between the gliptins as shown in (Figure S4 in the Supplementary Materials). However, they fit inside receptor binding site smoothly because of their optimum length. Some of them made HB interactions, sitagliptin—the second best docked—with PHE 295 also Anagliptin made two HBs with SER 203 and TYR 124. It is worth noting that (AChE) crystal structure shows one hydrogen bond with SER 125 in addition to the other non-bonding interactions. The remarkable finding is that the longer the designed structure the better to fit in the C shaped receptor pocket (Figure S5 in the Supplementary Materials).

**Figure 3:**
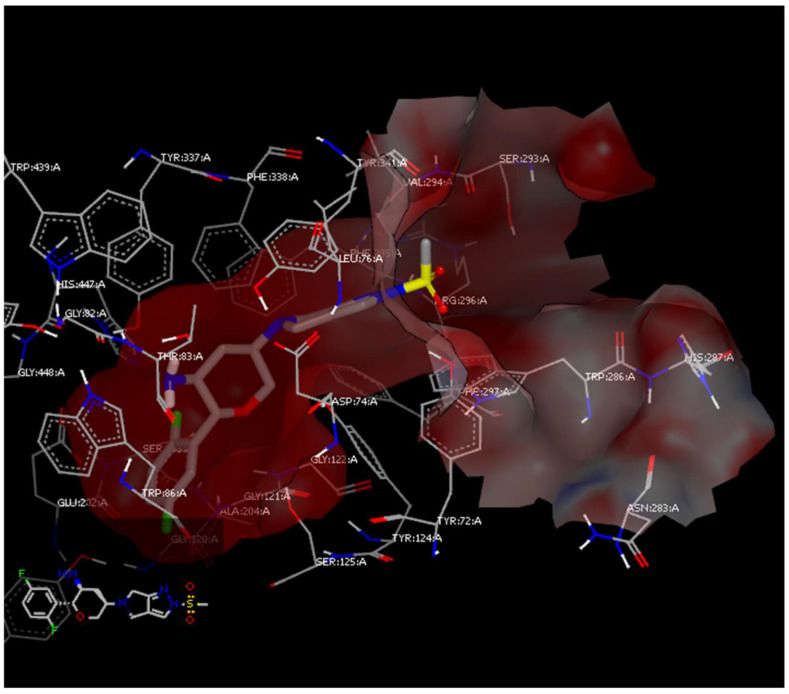
Predicted binding mode of OMR with AChE crystal structure.

OMR achieved a binding score lower than the ligand when docked with AChE (Figure 3). Yet, over observation of its proposed behavior with pocket, no polar interaction was found. The sulfonyl moiety with its two lone pairs is slightly protruded in the pocket center with no interactions with the surrounded amino acids. On the other point, deep inside binding site the benzene moiety with two fluorine atoms made a quite lean fitting. No hindrance was noticed between the conformer and adjoining residues in binding site.

It is worth noting that trelagliptin failed in achieving acceptable docking result with AChE as shown as N/A score in Table 1. Although the structural similarity between trelagliptin and alogliptin with only one fluorine atom as the difference between the two structures, alogliptin showed a perfect fit while trelagliptin did not which is attributed to the extra fluorine atom. The fluorine atom in trelagliptin hindered its fitting inside the pocket. Meanwhile, Alogliptin is fitted well forming two hydrogen bonds with TYR 124 and SER 203, plus the fitting within narrow bridge in receptor (Figure S6 in the Supplementary Materials). Although adding such a fluorine atom enhanced its anti-diabetic activity, on the other side, it seems that it decreases its neuroprotection.

#### 3.1.4. Comparative Docking Study of Gliflozins with A2AAR and AChE Receptors

A computational comparison study of 11 SGLT-2 inhibitors (Table 1) was carried out after docking with A2AAR and AChE receptors. SGLT2 inhibitors with adenosine receptor of Parkinson’s disease are found to bind with different style in comparison with the ligand as in (Figure S7 in Supplementary Materials). The ligand performs two hydrogen bonds with SER 253 on while gliflozins such as ipragliflozin and dapagliflozin made hydrogen bond on the counter side in the pocket with SER 67. Yet, both two binding behaviors are fitted within the pocket with other hydrophobic interactions. While with AChE, SGLT2 inhibitors form hydrogen bonds mainly because of the sugar part in mainly with SER 125 initiating the bonding mechanism then each structure according to its length continue to fit in the C-shaped pocket structure of the receptor. The remarkable finding that the docking scores of gliflozins and gliptins are much higher than those of the co-crystalized ligand implementing the same molecular dynamics and docking algorism.

### 3.2. GLP-1 Concentration

**Figure 4:**
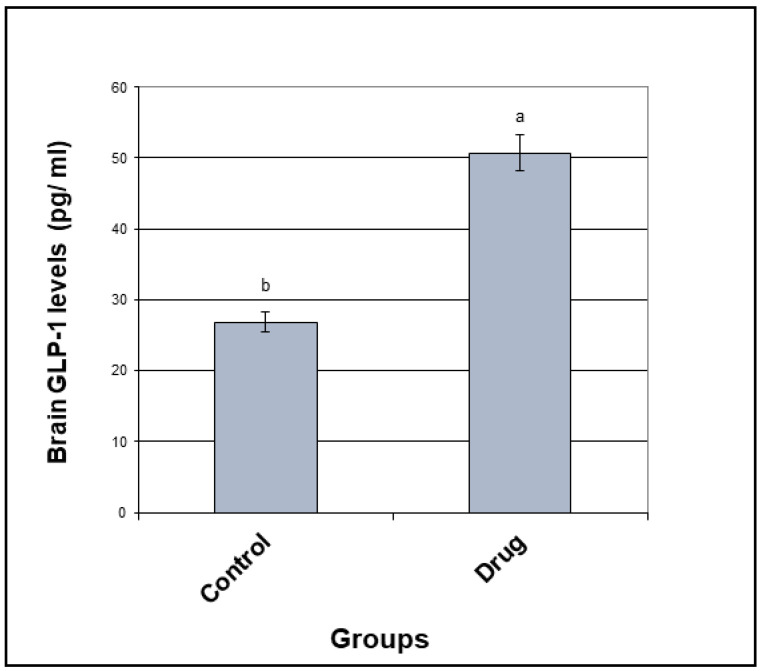
Statistical analysis of GLP-1 concentration in the drug group against the control group.

The brain glucagon-like peptide-1 (GLP-1) concentration was elevated by 1.9-fold following oral multiple doses of OMR (5 mg/kg/day, p.o. for 28 days) as compared to the control group (Figure 4). Concentration of GLP-1 is the drug group was 50.72 pg/mL ± 2.04 (SEM) while it was found to be 26.87 pg/mL ± 0.649 (SEM) in the control group which means more insulin sensitivity in the brain. It is becoming obvious: brain is an insulin sensitive organ that controls many metabolic signal cascades. Constant ATP supply in brain needs constant glucose uptake, which it turns controls general brain energetics. That is why the need for insulin with high sensitivity in the brain emerges as a preventing factor for neurodegenerative disorders and that was achieved by OMR. In addition, controlling blood glucose levels by OMR is in direct relation with better brain activities and cognitive functions.

### 3.3. BBB Crossing After 28 Days of Multiple Doses

A liquid chromatography tandem mass spectrometry method was developed for determination of OMR in rats’ plasma (10–3100 ng/mL) and rats’ brain tissue (15–2900 ng/mL) using liquid–liquid extraction. Alogliptin (ALP) was chosen to be the internal standard (IS) in this study due to its LogP value of 1.1, which is very close to the LogP of OMR. Multiple reaction monitoring (MRM) of the transition pairs of *m*/*z* equals 399.2 to 153.0 for OMR and *m*/*z* equals 340.2 to 116.0 for ALP was used in positive electro spray ionization (ESI) mode (Figure 1). Only one article that was published (by Ayoub et al.) considered the bioanalysis of OMR in rats’ brain tissue [17]. However, that method [17] used only direct precipitation with acetonitrile as an extraction technique for OMR. The lower limit of quantification (LLOQ) was 50 ng/mL [17], which is considered very high compared to the LLOQ in the present work (15 ng/mL). It is worth noting that the reported method [17] was not applicable in the current investigation that detected mean OMR brain tissue concentration of 543.56 ng/mL after considering the dilution factor of 10. The measured concentration before the dilution factor was 54.3 ng/mL, which is almost, equal to or very close to the LLOQ value of 50 ng/mL by Ayoub et al. method [17]. While that problem was solved by the described enhanced liquid–liquid extraction that showed LLOQ of 15 ng/mL which is suitable and applicable for the measured concentration after 28 days of multiple doses. Not only the extraction with ethyl acetate lowered the LLOQ but also using acetonitrile as the solvent for the IS decreased the emulsion between the matrix and the extracting solvent layers as the major advantages for the described method rather than the reported one [17].

It is worthy to mention that the reported bioanalytical method [17] that was published (by *Ayoub et al.*) included only single dose study of OMR after only 1 h of administration to rats while the current work included 28 days multiple doses study that simulate the long-term use of the drug in human consumption. Moreover, the current work included determination of GLP-1 concentration in brain tissue after 28 days multiple doses of OMR that showed a significant difference which is compatible with the findings of enhanced BBB crossing activity after the multiple doses. Furthermore, a part of the underlying work covered the comparative docking study, which was not included by any means in reference 17. Usually, studying multiple doses is preferred to a single dose to show the cumulative effects of the drug after metabolism and long-term exposure.

It is worth noting that the described extraction method was a slightly modified method applicable on both rats’ brain tissue and rats’ plasma samples and it had been firstly described and used for human plasma samples by Addy and Tatosian et al. [10,11,12,13]. Extraction of OMR from both rats’ plasma and rats’ brain tissue samples was successfully achieved with ethyl acetate as the extracting solvent after adding 1N sodium carbonate to enhance the drug migration, while choosing acetonitrile as the diluent solvent for the IS effectively decrease the emulsion between the layers in the liquid–liquid extraction followed by vacuum evaporation till dryness and reconstitution. Improved extraction was effectively attained after using acetonitrile as the diluent solvent for the IS decreasing the emulsion formed due to the addition of the aqueous immiscible organic solvent ethyl acetate [31]. Therefore, using an adequate mixture of the extracting solvent with high volume of acetonitrile; leads to decreasing the formed emulsion, and therefore allow the precise withdrawing of up to 1.3 mL from the upper pure organic layer.

Working on finding a suitable extraction method for both plasma and brain samples was a challenge in the current investigation. Although literature review showed the use of direct precipitation technique [16,17], the preliminary investigations in the described current research—using liquid–liquid extraction technique—showed clear enhanced results. In the previously published article [14], a mixture of diethyl ether and tertiary butyl methyl ether was used for the optimum extraction of OMR from human plasma. We tried the extraction of OMR from rats’ plasma using the same mixture [14] and it was successful because of that the rats’ plasma samples are less complicated matrix in comparison to human plasma samples but that was not the case in case of brain tissue samples. The main aim of the work was investigating OMR ratio (brain/plasma) applying the same extraction procedure to eliminate the error sources in calculations and partition coefficient. Moreover, many organic solvents had been tried before by Ayoub et al. in a previously published article [15] dealing with liquid–liquid extraction of OMR that included dichloromethane, hexane, ethyl acetate while all those extracting solvents showed less extracting power than ether without alkalinization. It is worthy to mention that when applying the method described in many articles (dealing with human plasma) based on ethyl acetate and alkalization with sodium carbonate [10,11,12,13], it showed the best results for both plasma and brain tissue. In spite of ethyl acetate is partially soluble in water, and therefore, it will extract all the nonpolar compounds and some polar metabolites, and the supernatant will contain a considerable portion of water, making it more tedious to evaporate it; in the current investigation, it showed the best recoveries especially for brain tissue samples after alkalinization.

**Figure 5:**
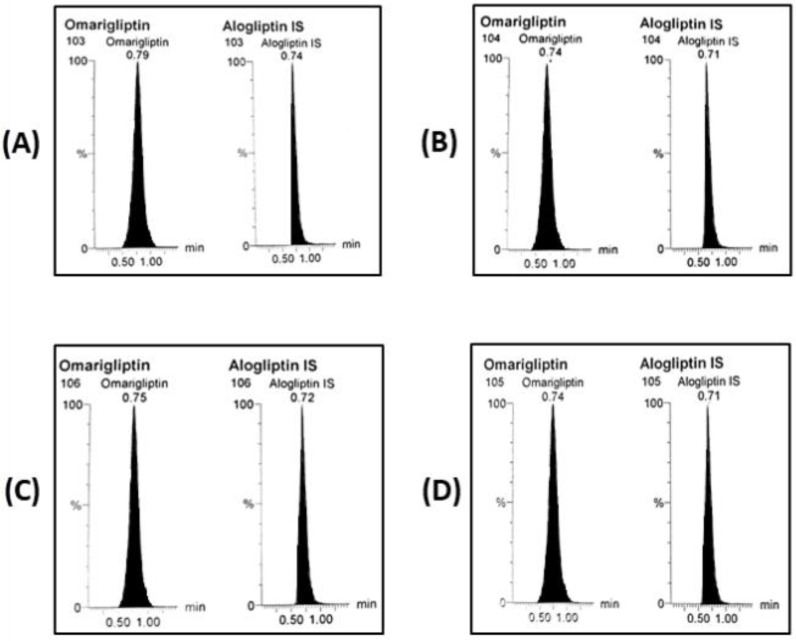
MRM chromatograms of OMR and IS (**A**) LLOQ plasma sample, (**B**) LLOQ brain homogenate sample, (**C**) biological plasma sample after 28 days multiple doses of OMR (5 mg/kg/day, p.o.), and (**D**) biological brain homogenate sample after 28 days multiple doses of OMR (5 mg/kg/day, p.o.).

Validation results were all satisfactory. Calibration (10–3100 ng/mL for rats’ plasma and 15–2900 ng/mL for brain homogenate), and full validation results were acceptable with FDA bio-analytical guidelines [30]. Appropriate method selectivity from six different batches of blank rats’ plasma and blank brain homogenate was designated where no notable interference was detected among the MRM channels in blank, zero samples with IS and reasonable outcomes at the LLOQ level (Figure 5A,B) and no notable carry over was observed as carry over percent was found to be 9.6% after plasma samples while it was found to be 8.1% after brain homogenate samples. The equations of the rats’ plasma and brain homogenates’ calibration curves were (y = 0.0103x + 0.0349, r = 0.9995) and (y = 0.0073 + 0.0309, r = 0.9997), respectively. Accuracy (*n*=5) and precision (*n*=15) were within ± 20% as shown in (Table 2). For the rats’ plasma method, extraction recovery was 74.62% for the LOQ and 80.13% for the HQC sample while they found to be (69.27%) and (72.53%), respectively for the brain homogenate method. Matrix factor signifying the effect of the matrix on the signal response and the ionization efficiency through matrix enhancement and/or suppression was calculated. It was found to be from 87.78% to 93.4% showing ion suppression for all concentrations of OMR in plasma while it ranged from 73.67% to 82.76% regarding the brain homogenate indicating more ion suppression. Dilution integrity results for rats’ plasma were acceptable after dilution of 3500 ng/mL 5 times (R% = 100.92 ± 6.48) and 10 times (R% = 93.48 ± 4.84) were acceptable. Dilution integrity results for rats’ brain homogenate were acceptable after dilution of 3000 ng/mL 5 times (R% = 90.32 ± 1.54) and 10 times (R% = 111.49 ± 12.87) were acceptable. Stability measurements stated under ‘methods’ indicated recoveries more than 80% (Table 3) that indicates OMR stability can be sustained through the sample treatment and storage. Working with high percent acetonitrile at the isocratic mode ensured the outcome of a very fast bio-analytical method suitable for the bio-analysis of more than 100 samples per day with enough selectivity, accuracy, and precision according to ICH guidelines. The authors used LC-MS/MS technique mainly as a ‘detection’ rather than ‘separation’ technique. Moreover, studying selectivity confirmed the absence of any interfering peaks from the studied different batches of blank matrix either for the rats’ plasma or rats’ brain tissue.

On day 28, OMR concentration in rats’ plasma was determined 2 h following the last dose (Figure 5C). It was found to be 1295.66 ± 684.63 ng/mL calculated from the bio-analysis regression equation. OMR passed through the BBB after the oral administration (Figure 5D) showing concentration of 543.56 ± 344.15 ng/g in brain tissue after considering the dilution factor of 10. The brain/plasma concentration ratio of 0.42 (543.56/1295.66) was used to predict the penetration power through the BBB after the multiple doses for 28 days. Results indicated that OMR passed through the BBB more effectively in the multiple dose study as compared to the previously published single dose study by the authors. Thus, the present study suggests potential repositioning of OMR as an antiparkinsonian agent.

## 4. Conclusions

As OMR crosses BBB successfully, its docking study results was more interesting than all the other docking studies for DPP-4 inhibitors and SGLT-2 inhibitors. Docking showed that OMR is perfectly fit into A2AAR binding pocket forming a distinctive hydrogen bond with threonine 256, besides other non-polar interactions inside the pocket suggesting the future of the marketed anti-diabetic drug (that cross BBB) as a potential anti-parkinsonian agent while OMR showed perfect fit inside AChE receptor binding site smoothly because of its optimum length that suggests also possible future repurposing as a neuroprotective agent against Alzheimer disease with the two fluorine atoms that enables quite lean fitting. Moreover, oral multiple doses of OMR (p.o, 5 mg/kg/day for 28 days) showed 1.9 times increase in brain GLP-1 concentration in comparison to the control group which means more insulin sensitivity in the brain. It is becoming obvious: brain is an insulin sensitive organ that controls many metabolic signal cascades. Constant ATP supply in brain needs constant glucose uptake, which it turns controls general brain energetics. That is why the need for insulin with high sensitivity in the brain emerges as a preventing factor for neurodegenerative disorders and that was achieved by OMR. In addition, controlling blood glucose levels by OMR is in direct relation with better brain activities and cognitive functions. Finally, as per FDA guidelines, bio-analysis of OMR using LC-MS/MS was established. Liquid–liquid extraction and reconstitution after vacuum evaporation was applied. The LLOQ in the present investigation is less than three times of the previous LLOQ stated by (Ayoub et al.). Enhancing the extraction techniques for new drugs enrich the literature and opens the door for more pharmacokinetic and bioequivalence studies. The brain/plasma concentration ratio of 0.42 (543.56/1295.66) was used to estimate the penetration power through the BBB after the multiple doses for 28 days. Results indicated that OMR crossed the BBB more effectively in the multiple dose study than the single dose study suggesting its possible repositioning as antiparkinsonian agent that will be of great impact for researchers concerned with neurodegenerative diseases.

## Figures and Tables

**Table 1:** Docking energy scores of 13 gliptins and 11 gliflozins with A2AAD and AChE receptors.

	SGLT2 Inhibitors	Score	DPP4 Inhibitors	Score
**AChE Receptors**	Ipragliflozin	−18.0826	Linagliptin	−16.7927
	Luseogliflozin	−17.4729	Sitagliptin	−14.6245
	Canagliflozin	−16.6481	Gemigliptin	−14.3007
	Dapagliflozin	−16.4979	Anagliptin	−14.2372
	Empagliflozin	−15.7074	Gosogliptin	−13.9488
	Sotagliflozin	−15.5315	Teneligliptin	−13.7557
	Tofogliflozin	−15.1791	Saxagliptin	−13.2396
	Ertugliflozin	−14.4763	Omarigliptin	−13.1468
	Sergliflozin	−13.4309	Alogliptin	−13.1226
	Remogliflozin	−12.2717	Evogliptin	−13.0186
	Ligand	−9.8658	Vildagliptin	−12.727
			Dutogliptin	−12.1961
			Ligand	−11.0175
			Trelagliptin	N/A
**A_2A_ AR Receptors**	Canagliflozin	−13.5012	Linagliptin	−11.6503
	Empagliflozin	−13.0962	Alogliptine	−11.14
	Ertugliflozin	−12.9675	Ligand	−10.7851
	Luseogliflozin	−12.2521	Trelagliptin	−10.778
	Ipragliflozin	−12.1433	Sitagliptin	−10.3922
	Dapagliflozin	−12.0988	Tenegliptin	−10.2282
	Sotagliflozin	−11.9531	Gosogliptin	−9.8775
	Tofogliflozin	−11.2769	Evogliptin	−9.8116
	Sergliflozin	−11.2085	Geminigliptin	−9.7432
	Ligand	−10.7851	Anagliptin	−9.2179
	Remogliflozin	−8.7495	Vildagliptin	−9.0521
			Dutogliptin	−8.5373
			Omarigliptin	−8.4469
			Saxagliptin	−8.3109

**Table 2:** Accuracy and precision results for OMR determination by the proposed LC-MS/MS.

Accuracy and Precision	Parameters	LLOQ	LQC	MQC	HQC
Rats’ plasma intra-day	Mean of R%	118.72	99.47	91.43	103.42
	Bias	18.72	−0.53	−8.57	3.42
	S.D.	13.03	10.95	7.21	8.76
	C.V. (%RSD)	10.97	11.01	7.89	8.47
Rats’ plasma inter-day	Mean of R%	99.81	101.75	91.09	101.35
	Bias	−0.19	1.75	−8.91	1.35
	S.D.	17.78	9.60	6.68	8.24
	C.V. (%RSD)	17.81	9.43	7.33	8.13
Rats’ brain intra-day	Mean of R%	86.99	87.96	110.25	109.62
	Bias	−13.01	−12.04	10.25	9.62
	S.D.	13.29	10.25	5.67	5.79
	C.V. (%RSD)	15.28	9.62	5.14	5.29
Rats’ brain inter-day	Mean of R%	83.23	90.61	99.80	107.24
	Bias	−16.77	−9.39	−0.20	7.24
	S.D.	14.04	8.18	9.26	5.59
	C.V. (%RSD)	16.88	9.03	9.28	5.21

Where S.D. is the standard deviation, C.V. is the coefficient of variation and %RSD is the percent relative standard deviation.

**Table 3:** Stability studies’ results for OMR determination by the proposed LC-MS/MS

Stability Studies	Parameters	Recoveries of LQC	Recoveries of HQC
Rats’ plasma	Auto-sampler stability	100.58 ± 0.41	100.58 ± 0.41
	Short-term stability	97.66 ± 1.66	109.83 ± 6.95
	Long-term stability	91.43 ± 6.06	105.17 ± 3.66
	Freeze–thaw stability	107.06 ± 4.99	111.14 ± 7.88
Rats’ brain	Auto-sampler stability	101.57 ± 1.11	104.97 ± 3.51
	Short-term stability	96.60 ± 2.40	106.40 ± 4.53
	Long-term stability	97.98 ± 1.42	112.86 ± 9.09
	Freeze–thaw stability	119.73 ± 13.95	119.57 ± 13.84

## Data Availability

Data is contained within the article and Supplementary Materials.

## Supplementary Materials

The following are available online. Figure S1: Docking score of OMR (Fragment No#2) relative to the receptor ligand, Figure S2: Predicted binding mode of alogliptin with 3PWH of A2AAR, Figure S3: Predicted binding mode of linagliptin with 3PWH of A2AAR, Figure S4: DPP-4 inhibitors with AChE receptor adapt different binding behaviors due to lack of structure similarity between the gliptins, Figure S5: The remarkable finding that the longer the designed structure the better to fit in the C shaped receptor pocket, Figure S6: Alogliptin is fitted well with AChE forming two hydrogen bonds with TYR 124 and SER 203, plus the fitting within narrow bridge in receptor, Figure S7: Different binding behavior of SGLT2 inhibitors and the cocrystlized ligand (in pink) of 6f25 in PDB.

## Sample Availability

Samples of the compounds are not available from the authors.
