# Supplementary material for: Repurposing of Omarigliptin as a Neuroprotective Agent Based on Docking with A2A Adenosine and AChE Receptors, Brain GLP-1 Response and Its Brain/Plasma Concentration Ratio after 28 Days Multiple Doses in Rats Using LC-MS/MS
Source: Molecules. 2021 Feb 8;26(4):889. doi: 10.3390/molecules26040889 (PMC7915074; doi:10.3390/molecules26040889)
Supplement: Supplementary file 1 [file molecules-26-00889-s001.pdf]

molecules

MDPI

Supplementary material

	Molecule	(green icon)	(lock icon)	(flag icon)	VIDA ID	FRED Chemgauss4 score
1	Unnamed molecule 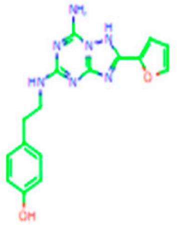	·	·	·	6	
2	Fragment 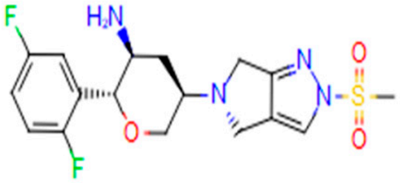	(green icon)	·	·	8	-8.3346

Figure S1. Docking score of OMR (Fragment No#2) relative to the receptor ligand.

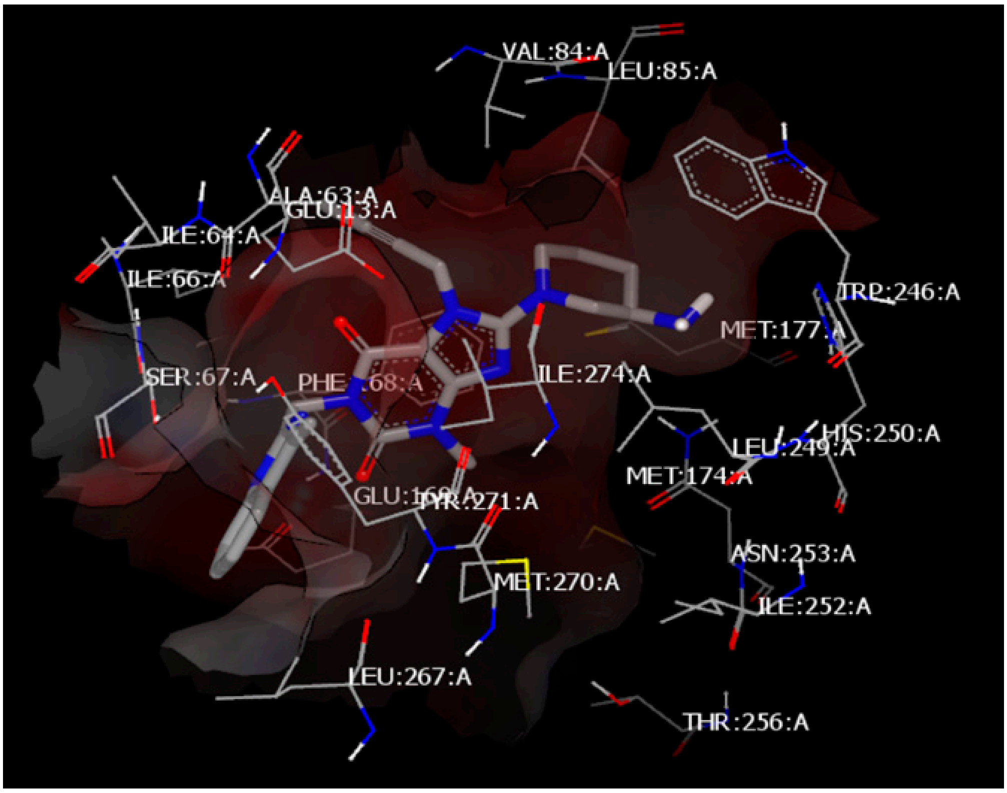

Figure S2. Predicted binding mode of alogliptin with 3PWH of A2AAR.

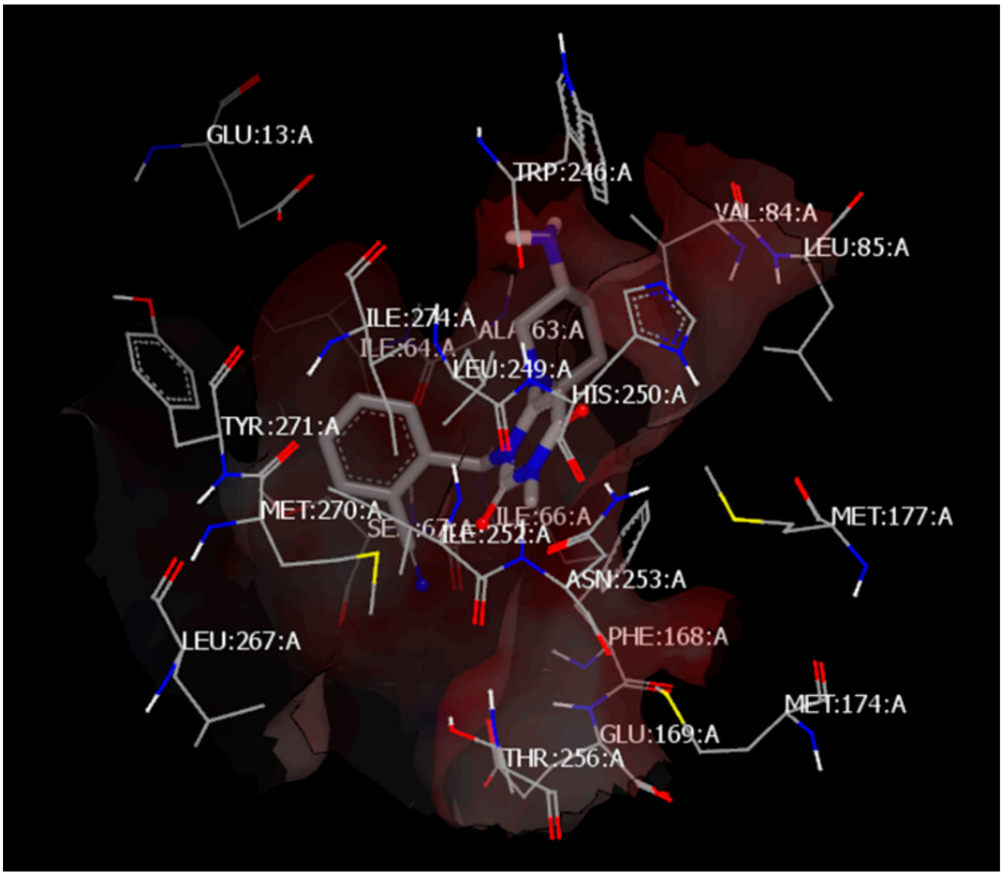

Figure S3. Predicted binding mode of linagliptin with 3PWH of A2AAR.

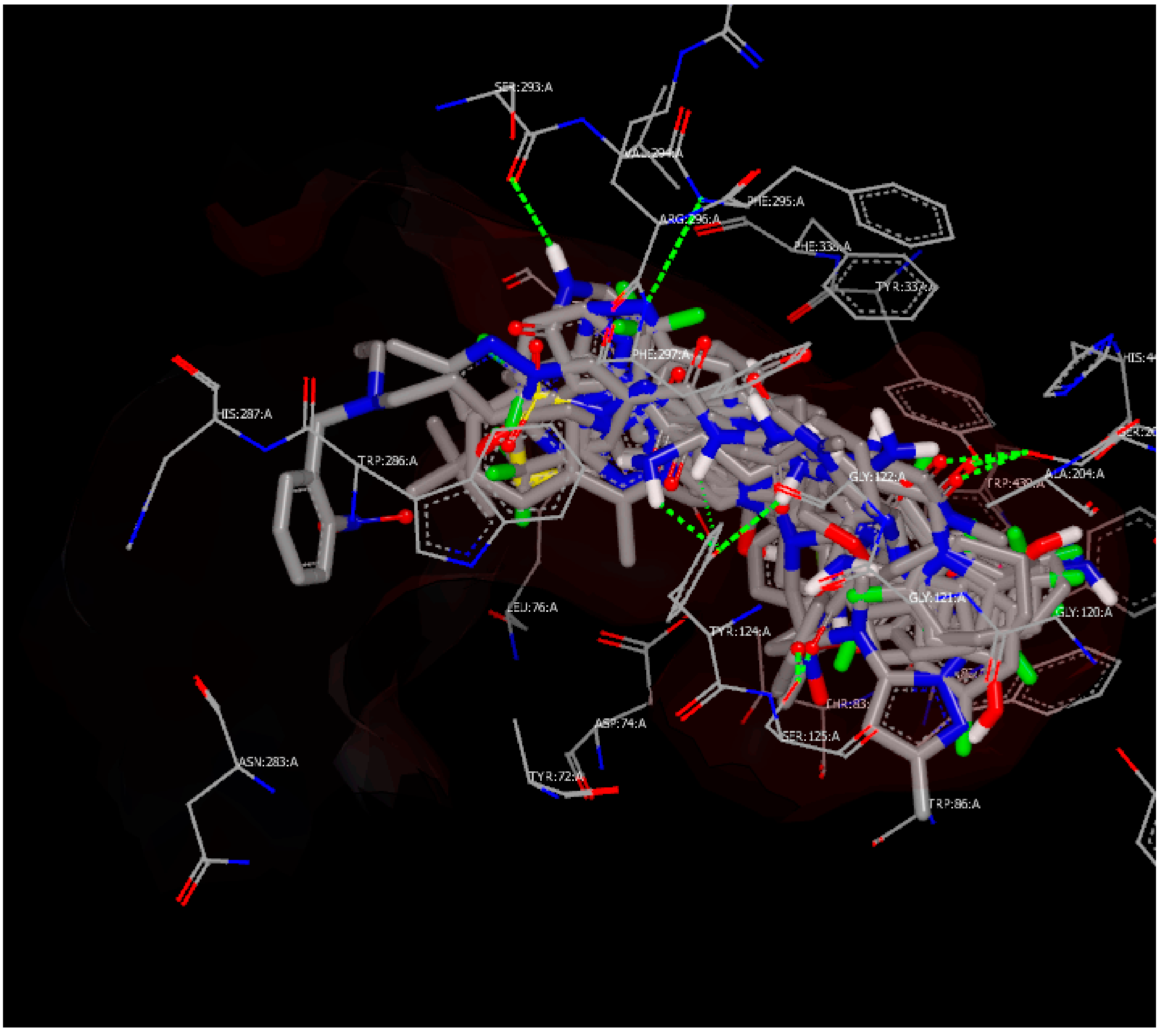

Figure S4. DPP-4 inhibitors with AChE receptor adapt different binding behaviors due to lack of structure similarity between the gliptins

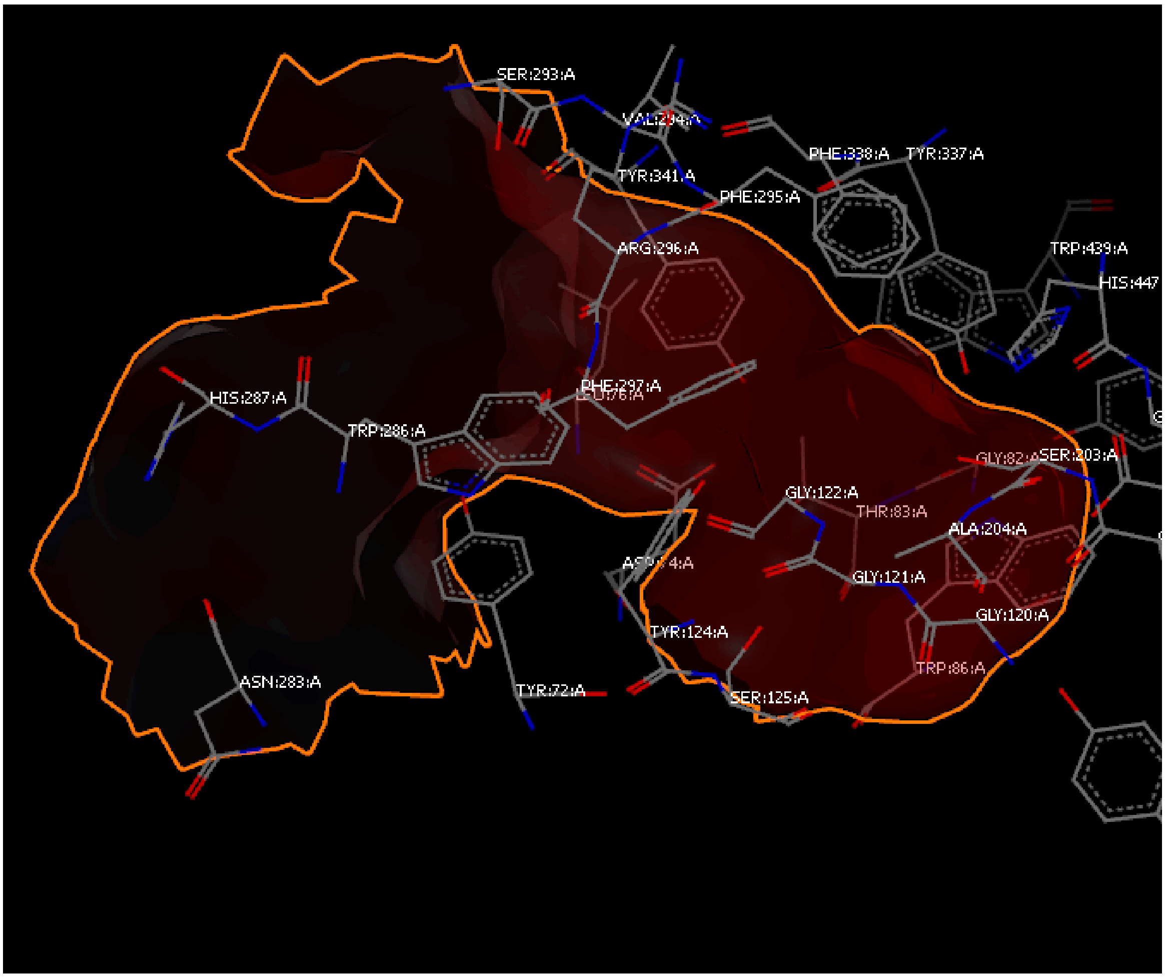

Figure S5. The remarkable finding that the longer the designed structure the better to fit in the C shaped receptor pocket

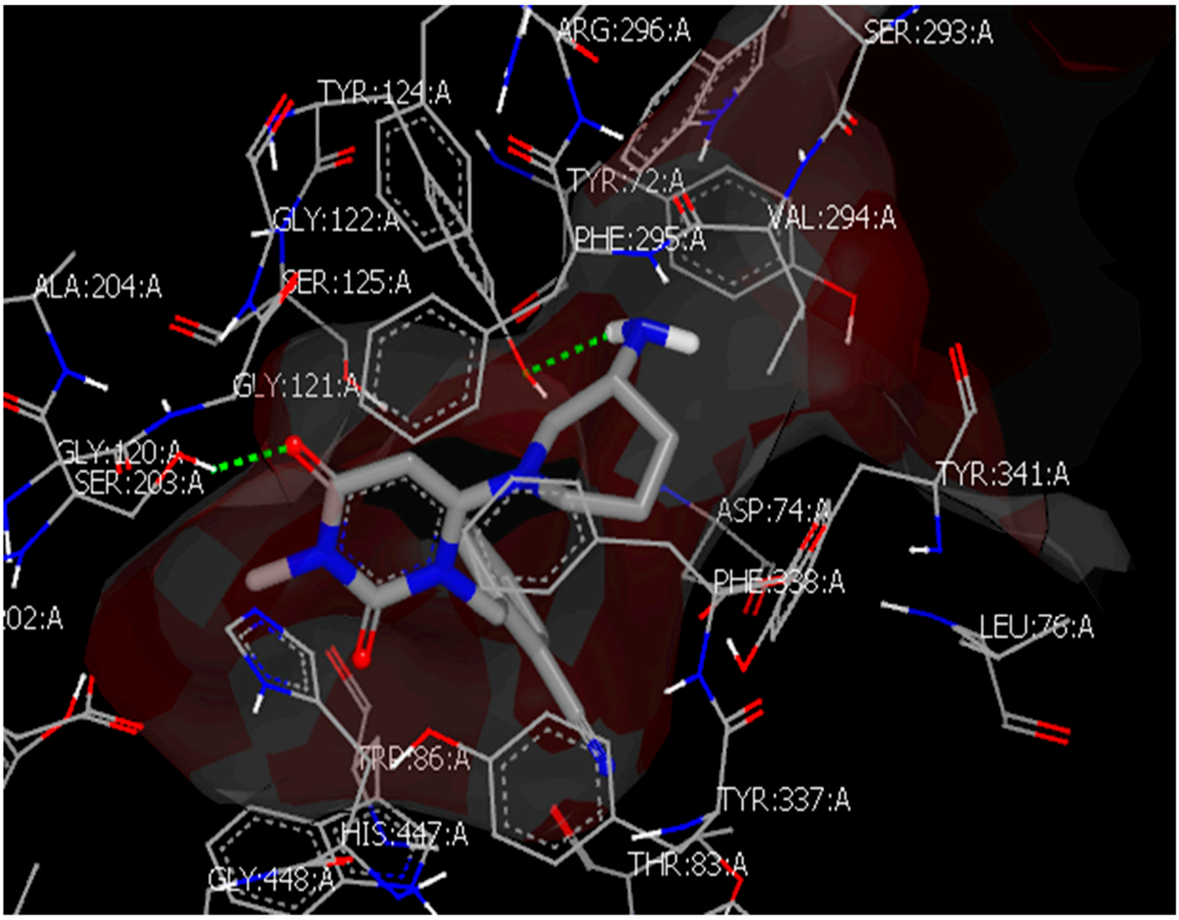

Figure S6. Alogliptin is fitted well with AChE forming two hydrogen bonds with TYR 124 and SER 203, plus the fitting within narrow bridge in receptor

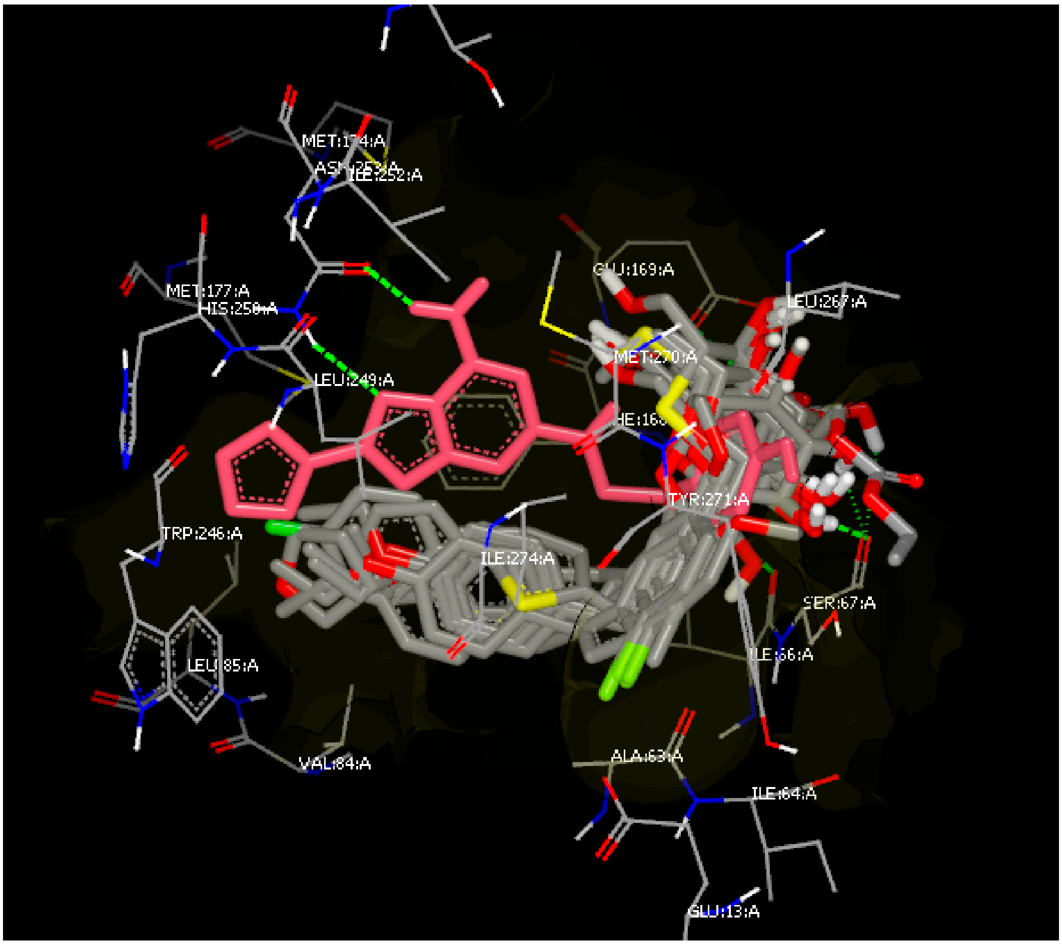

Figure S7. Different binding behavior of SGLT2 inhibitors and the cocrystlized ligand (in pink) of 6f25 in PDB.
